# Metabolic phenotyping of hand automatisms in mesial temporal lobe epilepsy

**DOI:** 10.1186/s13550-022-00902-1

**Published:** 2022-06-03

**Authors:** Jiajie Mo, Yao Wang, Jianguo Zhang, Lixin Cai, Qingzhu Liu, Wenhan Hu, Lin Sang, Chao Zhang, Xiu Wang, Xiaoqiu Shao, Kai Zhang

**Affiliations:** 1 Department of Neurosurgery, Beijing Tiantan Hospital, Capital Medical University, Beijing, China; 2 Department of Neurosurgery, Beijing Neurosurgical Institute, Capital Medical University, Beijing, China; 3 Pediatric Epilepsy Center, Peking University First Hospital, Peking University, Beijing, China; 4 Epilepsy Center, Peking University First Hospital Fengtai Hospital, Beijing, China; 5 Department of Neurology, Beijing Tiantan Hospital, Capital Medical University, Beijing, China

**Keywords:** Symptomatogenic zone, Metabolic network, Hand automatism, Temporal lobe epilepsy, Synchronization

## Abstract

**Purpose:**

Hand automatisms (HA) are common clinical manifestations in mesial temporal lobe epilepsy. However, the location of the symptomatogenic zone (EZ) in HA as well as the networks involved, are still unclear. To have a better understanding of HA underlying mechanisms, we analyzed images from interictal [^18^F] fluorodeoxyglucose-positron emission tomography (FDG-PET) in patients with mesial temporal lobe epilepsy (mTLE).

**Methods:**

We retrospectively recruited 79 mTLE patients and 18 healthy people that substituted the control group for the analysis. All patients underwent anterior temporal lobectomy and were seizure-free. Based on the semiology of the HA occurrence, the patients were divided into three subgroups: patients with unilateral HA (Uni-HA), with bilateral HA (Bil-HA) and without HA (None-HA). We performed the intergroup comparison analysis of the interictal FDG-PET images and compared the functional connectivity within metabolic communities.

**Results:**

Our analysis showed that the metabolic patterns varied among the different groups. The Uni-HA subgroup had significant differences in the extratemporal lobe brain areas, mostly in the ipsilateral supplementary motor area (SMA) and middle cingulate cortex (MCC) when compared to the healthy control group. The Bil-HA subgroup demonstrated that the bilateral SMA and MCC areas were differentially affected, whereas in the None-HA subgroup the differences were evident in limited brain areas. The metabolic network involving HA showed a constrained network embedding the SMA and MCC brain regions. Furthermore, the increased metabolic synchronization between SMA and MCC was significantly correlated with HA.

**Conclusion:**

The metabolic pattern of HA was most conspicuous in SMA and MCC brain regions. Increased metabolic synchronization within SMA and MCC was considered as the major EZ of HA.

## Highlights

1. Metabolic pattern analysis allowed allocation of the symptomatogenic zone (EZ) and brain network of hand automatisms (HA) in mesial temporal lobe epilepsy (mTLE).
2. The involved network of bilateral HA was larger than the unilateral one, probably due to the occurrence of contralateral dystonic posturing.
3. Increased metabolic synchronization within supplementary motor area (SMA) and middle cingulate cortex (MCC) regions were engaged in the representation and modulation of HA, suggesting these regions as the EZ for HA.

## Introduction

Mesial temporal lobe epilepsy (mTLE) is the most common form of focal seizures [1]. The ictal semiology of mTLE is characterized by oroalimentary and extremity automatisms, unilateral tonic, and dystonic posturing [2–6]. However, in some cases, versive head rotation or tilt can also be observed [4]. According to various reports, automatisms occur in two-thirds of individuals with focal seizures of mesial temporal lobe onset [7, 8]. The ictal hand automatisms (HA) are repetitive movements, as mostly seen in the ictal symptom, while HA can be manifested either unilateral or bilateral. However, among them, single motions such as grasping, fumbling, rubbing and flapping appear to be intentional and directed [9, 10]. The lateralization of HA has important value, concretely, Kotagal et al. showed that the accuracy of lateralization was higher when accompanied by the dystonic posturing of the opposite extremity [11], while in another study, 80% of the enrolled patients with unilateral HA reported ipsilateral lateralization [2].

Though HA are the exclusive symptoms of mTLE, they were detected in epilepsy originating from other areas of the brain [12]. There is evidence in the literature that associates the occurrence of HA in mTLE patients with the spread of epileptic discharge to extratemporal areas [13], which then strengthens the speculation that the symptomatogenic zone (EZ) is located in the extratemporal lobe. [^18^F]-fluorodeoxyglucose positron emission tomography (FDG-PET) imaging of brain glucose metabolism is a well-established and widely applicable technique for the lateralization and localization of epileptogenic foci in epileptic patients [14]. Interestingly, published data suggested that interictal hypometabolism topography was related to the neuronal networks involved by ictal discharge onset and spread pathways [15], whereas the seizure onset zone was confirmed by the stereoelectroencephalograhy (SEEG) [16]. Moreover, metabolic pattern analysis provided a principle approach to investigate the metabolic effects across a spatially distributed set of regions [17]. The last finding allows the automated quantitative analysis of glucose uptake in different brain regions and identified the neural networks.

Hence, it is of major importance to study the epilepsy network by elucidating the EZ of this semiology. Moreover, no data are confirming the association of EZ with HA. The present study aims to contribute to the identification of selective brain regions involved in patients with HA behaviors. We studied the whole-brain metabolic interictal FDG-PET pattern and synchronization of this common symptom in patients with mTLE and compared them with healthy individuals.

## Methods

### Patients

The study was approved by the Ethics Committee of the Beijing Tiantan Hospital, which was conducted under the Declaration of Helsinki. All participants had provided informed consent for the use of their medical records.

A total of 102 mTLE patients were examined at the Beijing Tiantan and Beijing Fengtai Hospitals from January 2015 to March 2019, among whom only 79 met the following inclusion criteria: (1) availability of high-quality neuroimaging data; (2) patients with no visible lesions on MRI except for hippocampal sclerosis; (3) patients that underwent anterior temporal lobectomy (ATL) and were seizure-free on follow up for at least 1 year after the surgery [18, 19]. The normal controls without history of neurological or psychiatric disorders were also recruited. Based on the semiology of the HA occurrence, the patients were divided into three subgroups: patients with unilateral HA (Uni-HA), with bilateral HA (Bil-HA) and without HA (None-HA). The mutual semiology included the common manifestations of mTLE, such as aura of fear, déjà vu, rising epigastric sensation, dialeptic seizures, oroalimentary and HA, as well as dystonic posturing, head deviation and version, and focal to bilateral seizures.

To localize the EZ, all patients were subjected to the evaluation of seizure semiology, electroencephalography (EEG), MRI and FDG-PET. The above examinations were completed within one week of presurgical evaluation. By studying the video EEG, two senior epileptologists confirmed the included semiology of HA: the patients with consistent HA at the beginning or during seizures, that lasted a minimum of 10 s were allocated into the HA group [3]. Regarding the divergent cases, the final decision was taken after the investigators reached a consensus. However, 2 cases were diagnosed with unilateral HA, in which contralateral automatisms were also manifested for a short time. Due to the raised disagreement and after group discussion, these two patients were classified as patients with bilateral involvement. On the occasion of not discovering the EZ in the non-invasive phase, SEEG was implanted to indicate the ictal pattern. The border of EZ was then delineated, and the minimum cortical resection was assessed [20–22].

### Neuroimaging data acquisition

All participants underwent T_1_-weighted magnetization prepared rapid acquisition gradient echo (T_1_WI MPRAGE), T_2_-weighted fluid-attenuated inversion recovery (T_2_WI FLAIR), and interictal FDG-PET [23, 24]. Structural MRIs were acquired for all the participants using a 3 T Siemens Verio scanner, including the T_1_WI MPRAGE sequence [repetition time (TR) = 2300 ms, echo time (TE) = 2.53 ms, flip angle = 12°, slice thickness = 1 mm, no gap, voxel size = 1.0 mm × 1.0 mm × 1.0 mm], and axial T_2_WI FLAIR sequence (TR = 7,000 ms, TE = 80 ms, flip angle = 12°, slice thickness = 1 mm, no gap, voxel size = 1.5 mm × 1.5 mm × 1.5 mm).

FDG-PET scans were obtained in the interictal phase. Moreover, the FDG-PET examinations were performed under standard resting conditions using the GE Discovery PET-CT system (matrix = 192 × 192, slice thickness = 3.27 mm). Briefly, the patients were reposed quietly in a dimly lit room for the following 40 min after the intravenous administration of ^18^F-FDG at a mean dose of 310 MBq/70 kg body weight. For the FDG-PET data reconstruction, we used the ordered subset expectation maximization algorithm (16 subsets and 6 iterations). Thereafter, any eventual attenuation in the reconstructed images was corrected with the use of a CT transmission scan. None of the patients had clinical seizures 6 h before or during the FDG-PET scan. Interictal FDG-PET scans were performed on all participants using similar protocols.

### FDG-PET data preprocessing and analysis

FDG-PET images were processed by MATLAB 2018a (The MathWorks, Natick, Massachusetts, USA) using the Statistical Parametric Mapping (SPM) toolbox, version 12 (Wellcome Department of Imaging Neuroscience, London, UK, https://www.fil.ion.ucl.ac.uk/spm/) [25]. Initially, the FDG-PET images were linearly co-registered with their respective T_1_-weighted MRI scans and then a spatial normalization of the obtained images was performed employing the Montreal Neurological Institute (MNI) template. To enhance the signal-to-noise ratio of these images, an 8-mm full width at half maximum Gaussian kernel was applied to smooth the FDG-PET images. For the reason that the low spatial solution of PET images may affect the quantitative analysis and visual interpretation, we applied partial volume effects (PVE) correction with the 3-compartmental voxel-wise Müller-Gärtner method. Thus, we increased the correspondence of the measured signal with the true regional tracer uptake. Importantly, it used segmentation of tissue compartments [gray matter (GM), white matter (WM), and cerebrospinal fluid] to correct the PET GM signal for spill-in effects from surrounding tissues, typically WM signals [26]. Furthermore, the intensity was normalized by separating the FDG-PET images activity from the global mean.

Metabolic pattern analysis was performed for the Uni-HA, Bil-HA and None-HA subgroups, and healthy controls using a voxel-based two-sample Student’s *t*-test for the comparison of the preprocessed FDG-PET image of each patient with the control database. In the statistical analysis were used only these voxels with > 30% of the maximum value (mask with a threshold of 0.3) reduce the background activity. The SPM maps threshold was corrected using *P* < 0.05 for multiple comparisons with the false discovery rate (FDR) method, whereas was applied a cluster threshold of 100 voxels [27, 28]. Metabolic synchronization calculations were retrieved by the normalization of the average FDG uptake values across the voxels. Each voxel was comprised of several distinct regions, thereby yielding a single uptake value for each region in every individual. The regions were selected from the Automated Anatomical Labeling atlas 3 (AAL3, https://www.oxcns.org/aal3.html) [29] and the metabolic values of 170 regions were recorded. The correlation between potentially significant brain regions concerning the HA was further calculated [17].

### Statistical analysis

The distributions of clinical data were analyzed by application of descriptive statistics. The normality of data distribution was assessed using the Lilliefors test, while the continuous variables were compared using either Student’s *t* test or Mann–Whitney *U-*test as appropriate. The distribution of categorical variables was compared using the Chi-squared (*χ*^*2*^) test. The obtained data with a normal distribution were expressed as mean ± standard deviation (SD), while the non-normal distributions were expressed as median and quartile. Moreover, the Pearson or Spearman correlation coefficients were calculated between significant brain regions. Statistical significance was set at a 5% level.

## Results

### Patient characteristics

Tables 1 and 2 illustrate the accumulated demographic details of the participants with left- and right-sided EZ, respectively.

**Table 1:** Demographic and clinical features of the patients with left-sided EZ

	Uni-HA	Bil-HA	None-HA	*P* value
Sex (M/F)	10/9	4/8	5/5	0.614
Frequency (*n*/month, median)	10	6	8	0.282
Epilepsy duration (years)	14.14 ± 7.81	14.00 ± 9.00	8.60 ± 4.97	0.155
Surgery age (years)	27.31 ± 6.19	27.75 ± 7.23	21.90 ± 9.91	0.137
Oroalimentary automatisms (*n*)	13	9	6	0.833
Head version (*n*)	5	1	2	0.529
Focal to bilateral seizures (*n*)	3	1	2	0.858

Uni-HA, Patients with unilateral hand automatisms (HA); Bil-HA, Patients with bilateral HA; None-HA, Patients without HA

**Table 2:** Demographic and clinical features of the patients with a right-sided EZ

	Uni-HA	Bil-HA	None-HA	*P* value
Sex (M/F)	5/10	7/2	7/7	0.104
Frequency (*n*/month, median)	7	5	5	0.464
Epilepsy duration (years)	12.13 ± 7.67	11.14 ± 9.43	11.50 ± 6.84	0.951
Surgery age (years)	26.46 ± 5.66	25.22 ± 7.18	23.71 ± 6.78	0.525
Oroalimentary automatisms (*n*)	8	6	6	0.596
Head version (*n*)	3	3	4	0.724
Focal to bilateral seizures (*n*)	2	2	2	0.865

Uni-HA, Patients with unilateral hand automatisms (HA); Bil-HA, Patients with bilateral HA; None-HA, Patients without HA

A total of 79 patients (males, *n* = 38) and 18 normal controls (male: *n* = 10, age: 22.6 ± 3.3 years) were enrolled for the conduction of the analysis. The mean age of the patients with mTLE (1) at the seizure onset was 13.42 ± 7.39 years (range: 0.5–32 years old), (2) at surgery 25.65 ± 7.09 years (range: 8–39 years old) and (3) at epilepsy duration of 12.22 ± 7.69 years (range: 1–33 years). The included patients were diagnosis as mTLE based on non-invasive evaluation and SEEG recording, and underwent surgery with seizure-free for an average of 20.77 ± 6.65 months (range: 12–36 months) follow-up.

Among the 79 patients with mTLE, 41 had a left-sided EZ, while the rest 38 had a right-sided EZ. In the subgroup with the left-sided EZ, 19 patients were assigned to the left Uni-HA subgroup, 12 patients to the left Bil-HA subgroup and 10 patients to the None-HA subgroup. Concerning the left Uni-HA subgroup, 17 patients reported ipsilateral HA at the EZ, while 2 patients had contralateral HA at the EZ. On the other hand, regarding the patients with right-sided EZ, 15 of them were allocated in the right Uni-HA subgroup, 9 in the right Bil-HA subgroup and 14 in the None-HA subgroup. However, all patients in the right Uni-HA subgroup manifested ipsilateral HA at the EZ. In addition to HA, oroalimentary automatisms, head and eye versions, and focal to bilateral seizures were the common semiologies observed in the recruited subjects. Interestingly, in patients with a left or right-sided EZ, we did not detect significant differences in the studied semiology among the left or the Uni-HA, Bil-HA and None-HA subgroups. Additionally, the analysis showed no significant differences in either sex, surgery age, epilepsy duration or seizure frequency among the subgroups.

### Intergroup comparison of the metabolic pattern

**Fig. 1:**
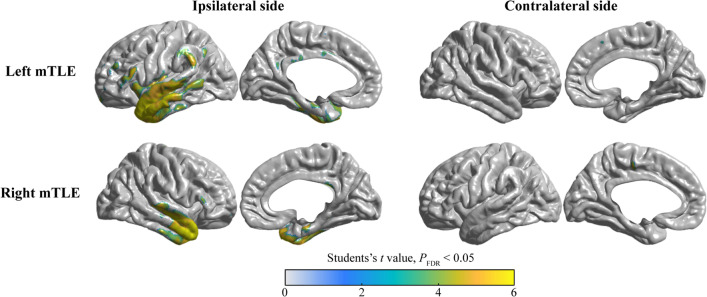
The metabolic pattern in patients with Uni-HA. Intergroup comparison of FDG-PET between left mTLE patients with healthy controls shows the hypometabolism in the left hippocampus, parahippocampal gyrus, insula, operculum, temporal neocortex, inferior parietal lobe, MCC and SMA (Top panel). However, right mTLE patients manifested a metabolic pattern, mainly focused on the right parahippocampal gyrus, temporal neocortex, operculum and right MCC (Bottom panel) The colorbar represents the Student’s *t* value with *P*_FDR_ < 0.05

The comparison between the Uni-HA subgroup and the control group showed that the patients with left mTLE showed hypometabolism in the left hippocampus, parahippocampal gyrus, insula, operculum, temporal neocortex, inferior parietal lobe, bilateral MCC, and SMA. Conversely, the patients with the right mTLE demonstrated a similar, but relatively limited metabolic pattern, as illustrated in Fig. 1.

**Fig. 2:**
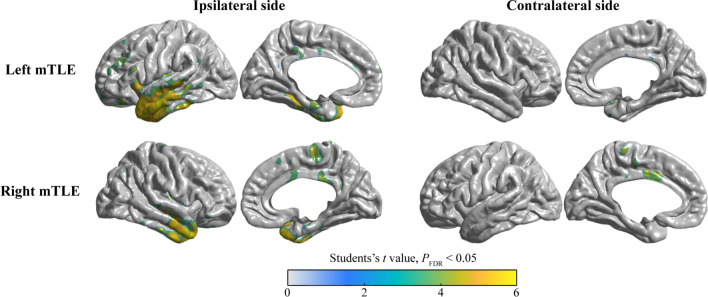
The metabolic pattern in patients with Bil-HA. Intergroup comparison of FDG-PET between left mTLE patients with healthy controls reveals the hypometabolism in the left hippocampus, parahippocampal gyrus, insula, frontal lobe, temporal neocortex, inferior parietal lobe and bilateral MCC (Top panel). However, right mTLE patients demonstrated a similar metabolic pattern, but the range was limited in the temporal neocortex with more widespread on the medial side, such as bilateral SMA and MCC (Bottom panel). The colorbar represents the Student’s *t* value with *P*_FDR_ < 0.05

The analysis of the data acquired from the comparison of the controls group with the Bil-HA subgroup showed a more obvious metabolic pattern than that of Uni-HA. Our findings indicated that patients with left mTLE had hypometabolism in the left hippocampus, parahippocampal gyrus, insula, frontal lobe, temporal neocortex, inferior parietal lobe and bilateral MCC. On the other hand, patients with right mTLE showed a similar but limited metabolic pattern in the temporal neocortex. Nevertheless, it was more widespread on the medial side, such as bilateral SMA and MCC (Fig. 2).

**Fig. 3:**
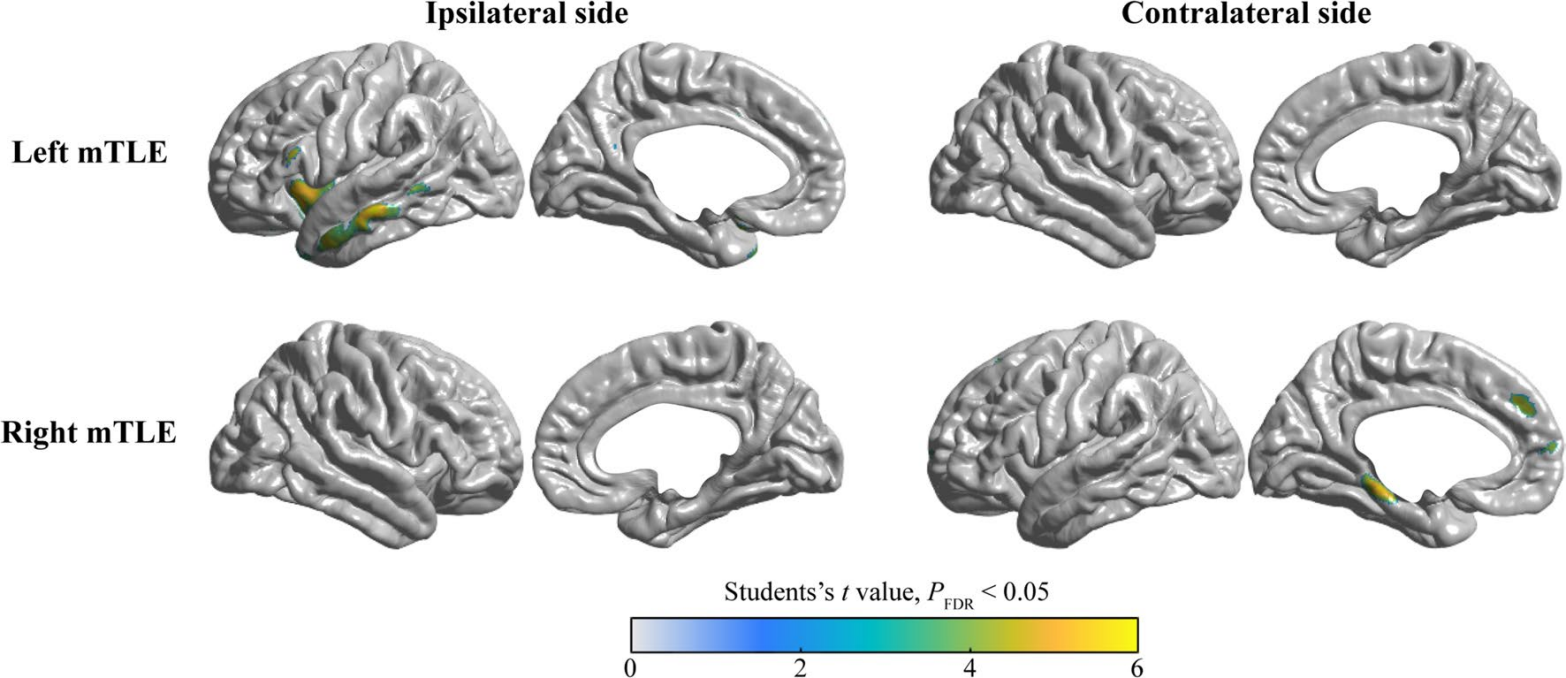
The metabolic pattern of patients with None-HA. The analysis of the comparison between the FDG-PET images of the left mTLE patients and those of the healthy participants demonstrated that the hypometabolism was found only in the left insula lobe and middle temporal gyrus. Moreover, it was not observed a significant metabolic pattern on the contralateral side (Top panel). On the contrary, the left parahippocampal gyrus and medial frontal lobe in the right mTLE patients showcased a subtle metabolic pattern (Bottom panel). The colorbar represents the Student’s *t* value with *P*_FDR_ < 0.05

In the None-HA subgroup, the metabolic pattern was less obvious than that of HA subgroups, when compared with the patients from the control group. Concretely, in individuals with left mTLE, hypometabolism was observed only in the left insula lobe and middle temporal gyrus, with no statistically significant metabolic pattern on the contralateral side. However, we detected a subtle metabolic pattern in patients with right mTLE, which was evident only in the left parahippocampal gyrus and medial frontal lobe (Fig. 3).

### Metabolic synchronization between SMA, MCC and hippocampus in different HA representations

We conducted a simultaneous metabolic pattern analysis of the Uni- and Bil-HA subgroups, which showed significant differences in the SMA and MCC. The retrieved findings suggested that SMA and MCC may serve as key regions for a brain network of HA, modulating the generation of HA. Concerning the patients from the groups Uni-HA, Bil-HA and None-HA, the correlation coefficient between SMA, MCC and hippocampus was calculated.

**Fig. 4:**
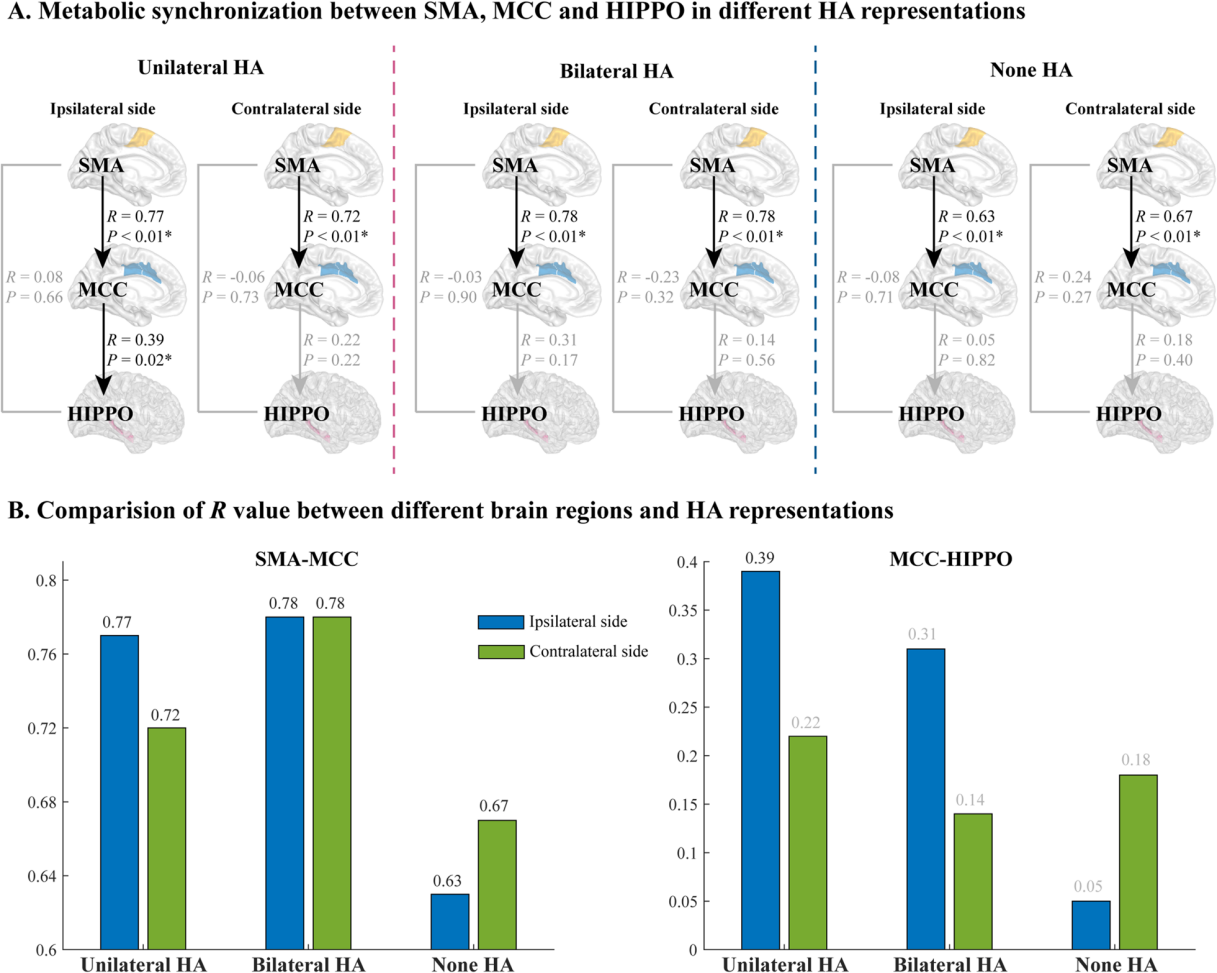
Metabolic synchronization between SMA, MCC and hippocampus. **A** Correlation of metabolic value between the SMA, MCC and hippocampus brain regions in Uni-HA, Bil-HA and None-HA subgroups. There was a significant correlation between SMA and MCC across all groups. The Uni-HA subgroup showed a significantly positive correlation between ipsilateral MCC and hippocampus, while there was no significant correlation between SMA and hippocampus on both sides across all subgroups. **B** Comparison of the *R* value among the 3 subgroups of patients. Following the correlation between SMA and MCC, the *R* value of the ipsilateral side was higher than this of the contralateral side in the Uni-HA subgroup. Moreover, it was equally high on both sides in individuals from the Bil-HA subgroup, while low in those who composed the None-HA subgroup. Regarding the correlation between MCC and hippocampus, the *R* value of the ipsilateral side was higher than this of the contralateral side in Uni- and Bil-subgroups, except for the None-HA subgroup in which the *R* value was found to be less

Our data revealed a high correlation between SMA and MCC among all the groups (Fig. 4A). In the Uni-HA subgroup, the Pearson *R* value of ipsilateral side (*R* = 0.77, *P* < 0.01) was higher than the contralateral side (*R* = 0.72, *P* < 0.01). Furthermore, patients from the Bil-HA subgroup, demonstrated high *R* value for both sides (both were *R* = 0.77, *P* < 0.01), whereas those patients from the None-HA subgroup had the lowest *R* value for both sides (*R* = 0.63, *P* < 0.01 and *R* = 0.67, *P* < 0.01) (Fig. 4B). For the ipsilateral MCC significant positive correlation was observed with hippocampus only in the Uni-HA subgroup (*R* = 0.39, *P* = 0.02), whereas no significant correlation between the SMA and the hippocampus in both sides across all subgroups was detected (Fig. 4).

## Discussion

mTLE is a common type of epilepsy caused by hippocampal sclerosis [30]. The typical semiology of mTLE includes focal seizures, which are included dialeptic seizures, automatisms, dystonic posturing and version, mostly accompanied by auras [2, 9, 31]. The semiology provides valuable information for the localization of the EZ. At present, the accumulated data suggest that both versions, as well as unilateral HA, are reliable signs of seizure lateralization [2, 11, 31]. Apart from the semiology of lateralization, its EZ and network are also widely studied and discussed [32]. Understanding the EZ and network is of utmost importance to interpreting the EEG and FDG-PET data as well as localizing the EZ. In the present study, the analysis of metabolic patterns provided evidence of the neuroimaging phenotyping of HA being focused on the SMA and MCC. It further presented the metabolic cortical network involving SMA and MCC as the EZ of HA. In addition, our data revealed that the increased metabolic synchronization within SMA and MCC region was engaged in the modulation of HA.

The probable role of the amygdala in the production of the fear aura has been reported in a previous study [5]. Also, Guedj and colleagues proposed that the parahippocampal region and lateral temporal cortex were possibly associated with the production of feelings of déjà vu [33]. In another study, the authors reported that the occurrence of oroalimentary automatism occurred due to the synchronization of the hippocampus and rolandic operculum [6]. Thus, we aimed to verify if similar mechanisms were involved in the development of HA.

### Symptomatogenic zone of hand automatisms

We analyzed the FDG-PET images of 79 mTLE patients. The ranges of the FDG-PET hypometabolism were determined via the ictal discharge generation and spread pathways [30, 34]. However, due to the large cohort study data, we could not flip them to one side for the group comparison of FDG-PET images. We classified the participants into 3 subgroups based on the involvement of either one, two or no hands. The metabolism pattern data showed better homogeneity in mTLE patients when compared with normal controls. The conducted analyses of metabolism revealed that there were significant differences in the SMA and MCC for both left and right-sided EZ. Interestingly we noticed two major findings: (1) the hypometabolic areas in the patients with a left-sided EZ were more extensive than in those with right-sided EZ, and (2) the involved brain areas associated with Uni-HA were more extensive than the areas in individuals with Bil-HA.

Multiple group comparisons showed that the SMA and MCC were more consistently involved in brain areas. Our findings suggested that the SMA and MCC are the brain regions that might be involved in the production of HA since these regions control movement. Moreover, our findings are consistent with reported data from previous studies concerning the vital role of extratemporal structures, particularly mesial frontal structures, in the development of extremity automatisms [13, 35, 36].

It is known that the SMA is located anterior to the primary motor area of the foot and limited by the below cingulate sulcus [37]. The responses elicited by this region are electrical stimulations that include movements of extremities [37] such as contralateral motor responses, although bilateral motor responses may also occur. Additionally, SMA plays a significant role in the physiology of the generation and control of action [38]. Since the SMA is involved in simple as well as complex motions, its function is not only presented in processing the motion but also has widespread connections with other regions of the motor cortex [39]. The connections of the SMA generally includes the ipsilateral motor cortex, cingulate gyrus and insula [40]. Meanwhile, it also provides a bond of the contralateral SMA with the basal ganglia [39, 41]. In regards to these data, our findings demonstrated that the unilateral and bilateral automatisms were related to the involvement of SMA.

Another brain area of great importance is the MCC, which is located on the medial side of the frontal lobe and in the middle of the cingulate gyrus. Besides, the relation of the MCC to movement, it is also known to be involved in multiple functions, such as feedback processing, pain, salience, action-reward association, premotor functions, and conflict monitoring [42]. Cortical labeling of the cervical or lumbar segment of the spinal cord showed that the arm movements were present in the cortex of the dorsal and ventral banks of the cingulate sulcus [43], which was similar to the position of the MCC in the current study. Lim et al. elicited upper and lower extremity movements by stimulating the SMA and MCC region [37]. Though MCC participates in a variety of functions, including complex hand movements, specifically reaching and grasping were elicited by the stimulation of MCC [44]. Similar to the SMA, the MCC has multiple functional connections with the motor cortex and the contralateral cingulate gyrus [45]. To the best of our knowledge, this is the first time that research data confirm that in mTLE patients, the SMA and MCC might be the EZ of HA.

### Network of hand automatisms

It is generally believed that the motion of the extremities is governed by the contralateral side of the functional areas. Though it is applicableapplies to the precentral gyrus, SMA and MCC [39], an electrical stimulation study showed that the stimulation of the SMA on the nondominant side induces bilateral extremity movements [46]. Herein, we investigated and analyzed whether HA was caused by the discharge to one side of the SMA and MCC. The analysis of FDG-PET images revealed that significant differences existed in patients with Uni- and bBil- HA in the SMA and MCC. However, the existence of strong functional connectivity of the SMA and MCC might cause easily spread of discharge to the opposite side. Subsequently, taking into consideration the obtained data, we suggested that the brain regions of SMA and MCC were involved in the generation as well as in the modulation of HA. Generally, automatisms are complex motions associated with several brain regions. However, similar to other automatisms [5, 6], HAs may be caused not only by the propagation of the epileptical discharge to a certain brain area but also by the synchronization among the multiple brain areas. Therefore, concerning future studies, it would be of major importance to study the possible synchronization between the mesial temporal lobe structures and the MCC or between the bilateral MCC and SMA.

Interestingly, our data showed that HA-related brain structures were almost similar in the studied groups of patients, despite the Uni- or Bil-HA, though the brain areas involved in bilateral automatisms were larger than those in the case of unilateral automatisms. This observed difference could be associated with the dystonic posturing during the Uni-HA. It is noteworthy to mention that several studies have confirmed that based upon the occurrence of dystonic posturing, basal ganglia structures are activated via the direct propagation of epileptic discharges [47, 48]. Furthermore, single photon emission computed tomography imaging in temporal lobe epilepsy has demonstrated a hyperperfusion in the ipsilateral putamen during the period of dystonic posturing [49]. Altogether, our data are in concurrence with the previous findings. However, dystonia involves a complex network leading to the need for more comprehensive elaboration in future studies.

### Limitations

Research on hippocampus with FDG-PET in the Alzheimer's Disease field failed to identify the differences between the groups probably due to poor spatial resolution. The authors employed the Jacobian information during spatial normalization to increase statistical power [50]. In the present study, we performed PVE correction with the voxel-wise Müller-Gärtner method to improve the detection of hypometabolic regions.

## Conclusions

The results of the present study proved that the EZ of HA was mainly located in the SMA and MCC brain region, with the involvement in both Uni- and Bil-HA. The strong functional connection between the bilateral SMA and MCC enabled the discharge to quickly spread to the opposite side. We assumed that the metabolic cortical network of SMA and MCC could be considered as a EZ of HA, while the increased metabolic synchronization within these two regions was engaged in the representation and modulation of HA.

## Data Availability

The data sets generated and analyzed in the course of the current study are available from the corresponding author on reasonable request.
